# Different prognostic impact of recurrent gene mutations in chronic lymphocytic leukemia depending on IGHV gene somatic hypermutation status: a study by ERIC in HARMONY

**DOI:** 10.1038/s41375-022-01802-y

**Published:** 2022-12-24

**Authors:** Larry Mansouri, Birna Thorvaldsdottir, Lesley-Ann Sutton, Georgios Karakatsoulis, Manja Meggendorfer, Helen Parker, Ferran Nadeu, Christian Brieghel, Stamatia Laidou, Riccardo Moia, Davide Rossi, Mark Catherwood, Jana Kotaskova, Julio Delgado, Ana E. Rodríguez-Vicente, Rocío Benito, Gian Matteo Rigolin, Silvia Bonfiglio, Lydia Scarfo, Mattias Mattsson, Zadie Davis, Ajay Gogia, Lata Rani, Panagiotis Baliakas, Hassan Foroughi-Asl, Cecilia Jylhä, Aron Skaftason, Inmaculada Rapado, Fatima Miras, Joaquín Martinez-Lopez, Javier de la Serna, Jesús María Hernández Rivas, Patrick Thornton, María José Larráyoz, María José Calasanz, Viktória Fésüs, Zoltán Mátrai, Csaba Bödör, Karin E. Smedby, Blanca Espinet, Anna Puiggros, Ritu Gupta, Lars Bullinger, Francesc Bosch, Bárbara Tazón-Vega, Fanny Baran-Marszak, David Oscier, Florence Nguyen-Khac, Thorsten Zenz, Maria Jose Terol, Antonio Cuneo, María Hernández-Sánchez, Sarka Pospisilova, Ken Mills, Gianluca Gaidano, Carsten U. Niemann, Elias Campo, Jonathan C. Strefford, Paolo Ghia, Kostas Stamatopoulos, Richard Rosenquist

**Affiliations:** 1 Department of Molecular Medicine and Surgery, Karolinska Institutet, Stockholm, Sweden; 2 Centre for Research and Technology Hellas, Institute of Applied Biosciences, Thessaloniki, Greece; 3 Department of Mathematics, University of Ioannina, Ioannina, Greece; 4 MLL Munich Leukemia Laboratory, Munich, Germany; 5 Cancer Genomics, School for Cancer Sciences, Faculty of Medicine, University of Southampton, Southampton, UK; 6 Institut d’Investigacions Biomèdiques August Pi i Sunyer (IDIBAPS), Barcelona, Spain; 7 Centro de Investigación Biomédica en Red de Cáncer (CIBERONC), Madrid, Spain; 8 Department of Hematology, Rigshospitalet, Copenhagen University Hospital, Copenhagen, Denmark; 9 Division of Hematology, Department of Translational Medicine, Università del Piemonte Orientale, Novara, Italy; 10 Division of Hematology, Oncology Institute of Southern Switzerland, Bellinzona, Switzerland; 11 Laboratory of Experimental Hematology, Institute of Oncology Research, Bellinzona, Switzerland; 12 Patrick G Johnston Centre for Cancer Research, Queen’s University Belfast, Belfast, UK; 13 Department of Internal Medicine—Hematology and Oncology, University Hospital Brno, Brno, Czech Republic; 14 Faculty of Medicine, Masaryk University, Brno, Czech Republic; 15 Central European Institute of Technology, Masaryk University, Brno, Czech Republic; 16 Hospital Clínic of Barcelona, Barcelona, Spain; 17 Universitat de Barcelona, Barcelona, Spain; 18 Cancer Research Center (IBMCC) CSIC—University of Salamanca, Salamanca, Spain; 19 Instituto de Investigación Biomédica (IBSAL), Salamanca, Spain; 20 Department of Hematology, University Hospital of Salamanca, Salamanca, Spain; 21 Hematology—Department of Medical Sciences, University of Ferrara, Ferrara, Italy; 22 Università Vita Salute San Raffaele and IRCCS Ospedale San Raffaele, Milano, Italy; 23 Department of Immunology, Genetics and Pathology, Uppsala University, Uppsala, Sweden; 24 Molecular Pathology Department, University Hospitals Dorset, Bournemouth, UK; 25 All India Institute of Medical Sciences (AIIMS), New Delhi, India; 26 Hospital Universitario 12 Octubre, Madrid, Spain; 27 Spanish National Cancer Research (CNIO), Madrid, Spain; 28 Haematology Department, Beaumont Hospital, Dublin, Ireland; 29 Hematological Diseases Laboratory, CIMA LAB Diagnostics, University of Navarra, Pamplona, Spain; 30 IdiSNA, Navarra Institute for Health Research, Pamplona, Spain; 31 HCEMM-SE Molecular Oncohematology Research Group, Department of Pathology and Experimental Cancer Research, Semmelweis University, Budapest, Hungary; 32 Central Hospital of Southern Pest—National Institute of Hematology and Infectious Diseases, Budapest, Hungary; 33 Clinical Epidemiology Division, Department of Medicine Solna, Karolinska Institutet, Stockholm, Sweden; 34 Molecular Cytogenetics Laboratory, Pathology Department, Hospital del Mar and Translational Research on Hematological Neoplasms Group, Hospital del Mar Research Institute (IMIM), Barcelona, Spain; 35 Department of Hematology, Oncology and Cancer Immunology, Charité-Universitätsmedizin Berlin, Corporate Member of Freie Universität Berlin, Humboldt-Universität zu Berlin, Berlin, Germany; 36 Department of Hematology, Hospital Universitari Vall d’Hebron (HUVH), Experimental Hematology, Vall d’Hebron Institute of Oncology (VHIO), Department of Medicine, Universitat Autònoma de Barcelona, Barcelona, Spain; 37 Service d’hématologie Biologique Hôpital Avicenne Assistance Publique des Hôpitaux de Paris, Bobigny, France; 38 Sorbonne Université, Service d’Hématologie Clinique, Hôpital Pitié-Salpêtrière, APHP, Paris, France; 39 Department of Oncology and Haematology, University Hospital and University of Zurich, Zurich, Switzerland; 40 Department of Hematology, INCLIVA Research Insitute, University of Valencia, Valencia, Spain; 41 Clinical Genetics, Karolinska University Hospital, Solna, Sweden

**Keywords:** Cancer genetics, Genetics research

## Abstract

Recent evidence suggests that the prognostic impact of gene mutations in patients with chronic lymphocytic leukemia (CLL) may differ depending on the immunoglobulin heavy variable (IGHV) gene somatic hypermutation (SHM) status. In this study, we assessed the impact of nine recurrently mutated genes (*BIRC3*, *EGR2*, *MYD88, NFKBIE*, *NOTCH1*, *POT1*, *SF3B1, TP53*, and *XPO1*) in pre-treatment samples from 4580 patients with CLL, using time-to-first-treatment (TTFT) as the primary end-point in relation to IGHV gene SHM status. Mutations were detected in 1588 (34.7%) patients at frequencies ranging from 2.3–9.8% with mutations in *NOTCH1* being the most frequent. In both univariate and multivariate analyses, mutations in all genes except *MYD88* were associated with a significantly shorter TTFT. In multivariate analysis of Binet stage A patients, performed separately for IGHV-mutated (M-CLL) and unmutated CLL (U-CLL), a different spectrum of gene alterations independently predicted short TTFT within the two subgroups. While *SF3B1* and *XPO1* mutations were independent prognostic variables in both U-CLL and M-CLL, *TP53*, *BIRC3* and *EGR2* aberrations were significant predictors only in U-CLL, and *NOTCH1* and *NFKBIE* only in M-CLL. Our findings underscore the need for a compartmentalized approach to identify high-risk patients, particularly among M-CLL patients, with potential implications for stratified management.

## Introduction

Chronic lymphocytic leukemia (CLL) is clinically heterogenous, ranging from an indolent condition without urgent need for treatment, to an aggressive disease characterized by rapid progression, resistance to therapy, and poor overall survival. The Rai and Binet staging systems are used in clinical practice to assess prognosis in patients with CLL [1, 2]. However, in early-stage disease, which constitute the great majority of new diagnoses, these systems are unable to predict which cases will progress to a more aggressive disease [3]. That said, there are a number of molecular markers with prognostic and/or predictive impact that should be assessed in all patients prior to treatment initiation [4, 5]. These include genomic aberrations detected by fluorescence in situ hybridization (FISH), such as del(17p), del(11q), trisomy 12 and del(13q), and the immunoglobulin heavy variable (IGHV) gene somatic hypermutation (SHM) status [6–9]. In addition, *TP53* sequence analysis is essential, as patients with *TP53* mutations, irrespective of the presence of del(17p), experience a more adverse outcome, even with targeted therapies, particularly in the relapsed/refractory setting [5, 10–12].

Over the past decade, next-generation sequencing (NGS) studies have led to the discovery of recurrently mutated genes in CLL, such as *NOTCH1*, *SF3B1*, *BIRC3*, *XPO1*, *POT1*, *NFKBIE* and *EGR2*, that are associated with poor clinical outcome [13–21]. On the other hand, recurrent mutations in *MYD88* have been correlated with a favorable outcome, though not conclusively [22, 23]. Based on these findings, several studies have devised prognostic models that incorporate clinicobiological factors as well as various combinations of recurrent gene mutations, aimed at improving risk stratification in CLL [24–26]. However, several questions remain unanswered. While *NOTCH1*, *SF3B1* and *BIRC3* mutations have been investigated in larger patient cohorts [17, 25, 27, 28], the prognostic role of the remaining gene mutations is less well studied. Furthermore, the frequencies and clinical impact of gene mutations differ between the poor-prognostic IGHV-unmutated (U-CLL) subgroup and the more favorable-prognostic IGHV-mutated CLL (M-CLL) subgroup [29–32]. In fact, in recent studies, a number of genetic markers were demonstrated to affect outcome in U-CLL and M-CLL differently, indicating that a more compartmentalized approach based on the IGHV gene SHM status might be necessary when developing prognostic models [31, 32].

In this study, we investigated the prognostic roles of nine genes recurrently mutated in CLL, as well as their relative impact in U-CLL and M-CLL, with a particular focus on early-stage patients and time-to-first-treatment (TTFT) as the primary clinical endpoint. This analysis was performed in a well-annotated series of pre-treatment samples from 4580 patients with CLL, to our knowledge the largest series analyzed thus far, consolidated in the context of a European multicenter effort coordinated by the European Research Initiative on CLL (ERIC) as partner of the HARMONY Alliance. Our novel data highlight that different spectra of genetic mutations predict clinical outcome in U-CLL and M-CLL, respectively, with *SF3B1* and *XPO1* mutations being the most significant prognostic markers irrespective of subgroup assignment.

## Materials and methods

### Patients

Our cohort included 4580 patients with CLL from 26 European centers (Supplementary Table S1). Only pre-treatment samples were analyzed; the median time from diagnosis to sample collection was 3 months (data available for 3991 samples). The majority of cases were non-trial patients from referral centers, while the cohort also included patients from the UK LRF CLL4 clinical trial [33] (*n* = 499). The median age at diagnosis was 64.6 years (interquartile range, 56.7–71.7 years). Clinicobiological characteristics for the cohort are shown in Table 1. All cases were diagnosed according to the iwCLL guidelines [4]. Informed consent was obtained according to the Helsinki declaration and the study was approved by the local Ethics Review Committees.

**Table 1:** Clinicobiological characteristics of the studied cohort.

	All cases	Binet A	Binet A M-CLL	Binet A U-CLL
Gender				
Male	2890 (63%)	1992 (59%)	1183 (58%)	708 (61%)
Female	1690 (37%)	1370 (41%)	862 (42%)	445 (39%)
Median age at diagnosis (years)	64.6	64.9	64.6	64.9
IGHV gene SHM status				
M-CLL	2454 (57%)	2045 (64%)		
U-CLL	1878 (43%)	1153 (36%)		
Unknown	239	164		
Recurrent aberrations^a^				
del(13q)	1856 (42%)	1465 (45%)	775 (57%)	302 (27%)
trisomy12	566 (13%)	380 (12%)	72 (5%)	212 (19%)
del(11q)	495 (11%)	277 (8%)	12 (1%)	225 (20%)
del(17p)	236 (5%)	148 (5%)	42 (3%)	78 (7%)
No recurrent abnormalities	1306 (29%)	1005 (31%)	469 (34%)	319 (28%)
Unknown	121	87	35	17
Binet stage				
A	3362 (74%)			
B	825 (18%)			
C	387 (8%)			
Unknown	6			
Treatment status^b^				
Treated	2680 (59%)	1570 (47%)	640 (32%)	875 (76%)
Untreated	1900 (41%)	1792 (53%)	1405 (68%)	278 (24%)

*SHM* somatic hypermutation, *M-CLL* CLL with mutated IGHV genes, *U-CLL* CLL with unmutated IGHV genes.

^a^According to the Döhner classification [6].

^b^At follow-up.

### Mutational analysis

All cases were assessed for mutations within the coding sequences of the *BIRC3*, *EGR2*, *MYD88, NFKBIE*, *NOTCH1*, *POT1*, *SF3B1, TP53*, and *XPO1* genes. The *ATM* gene was initially included, however, mutation data was only available for 3611 cases and considering the difficulties to interpret *ATM* mutation without germline DNA, it was excluded from further analysis. Mutational screening was performed by NGS for the majority of the cohort (80%), with targeted gene panels, covering all or hotspot exons of each gene, as the most frequent technique employed. Sanger sequencing was performed in 18% of cases and the remaining methods combined, mostly targeting hotspot mutations, accounted for 2% of analyses (Supplementary Table S1). For mutational data generated using NGS analysis, sequence alignment, annotation and variant calling were performed at each center using a ≥5% variant allele frequency (VAF) threshold to classify mutated cases. Variant filtering was performed by including nonsynonymous variants and small insertions/deletions within the coding sequences and by excluding variants with a population frequency >0.0001 in the gnomAD database unless variant was included as a somatic variant in the COSMIC database.

The mutation frequencies were compared in samples analyzed by NGS and Sanger sequencing, examining both hotspot mutations (exemplified by *SF3B1* p.K700E, *NOTCH1* p.P2514fs and *EGR2* p.H383N mutations) or entire genes without hotspot positions (exemplified by *TP53* and *XPO1*), without significant differences in mutation rates when comparing the methods (Supplementary Table 2).

IGHV gene SHM status, available for 4332/4580 (94.6%) patients, was determined using PCR amplification and sequence analysis of IGHV-IGHD-IGHJ gene rearrangements and employing a 98% identity cut-off to germline to define M-CLL (<98% identity) and U-CLL (≥98% identity) [9]. Chromosomal aberrations [data available for 4459/4580 (97.4%) cases] were detected using FISH with targeted probes for chromosomes 13q, 11q, 17p and 12 or Affymetrix 250K SNP-arrays and classified according to the Döhner hierarchical model [6].

### Statistical analysis

The chi-square test was employed for comparing the clinicobiological variables and *p* values were Yates corrected. Co-occurrence and exclusivity matrices were constructed using two-sided Fisher’s exact tests and *p* values were corrected using Benjamini–Hochberg for multiple testing. TTFT was available for 4543/4580 (99.2%) cases and calculated from the diagnostic date until date of first treatment; median follow-up was 3.6 years for the entire cohort and 6.5 years for untreated patients. Survival curves were constructed using the Kaplan–Meier method, pairwise comparisons using the Cox–Mantel log-rank test determined differences between subgroups and *p* values were adjusted using the Benjamini–Hochberg method. The Cox proportional hazards model was employed to assess the prognostic strength of each marker in multivariable analysis. Stepwise variable selection using Akaike’s information criterion was applied in the Cox regression model to examine the significant risk factors associated with TTFT, and the proportion of the explainable log-likelihood was used to examine their relative importance. All statistical analyses were performed using R (version 4.1.2) and R Studio software. Plots were created using ggplot2 v3.3.5, ComplexHeatmap v2.11.1 and G3viz packages [34].

## Results

### Frequency of recurrent gene mutations

**Fig. 1:**
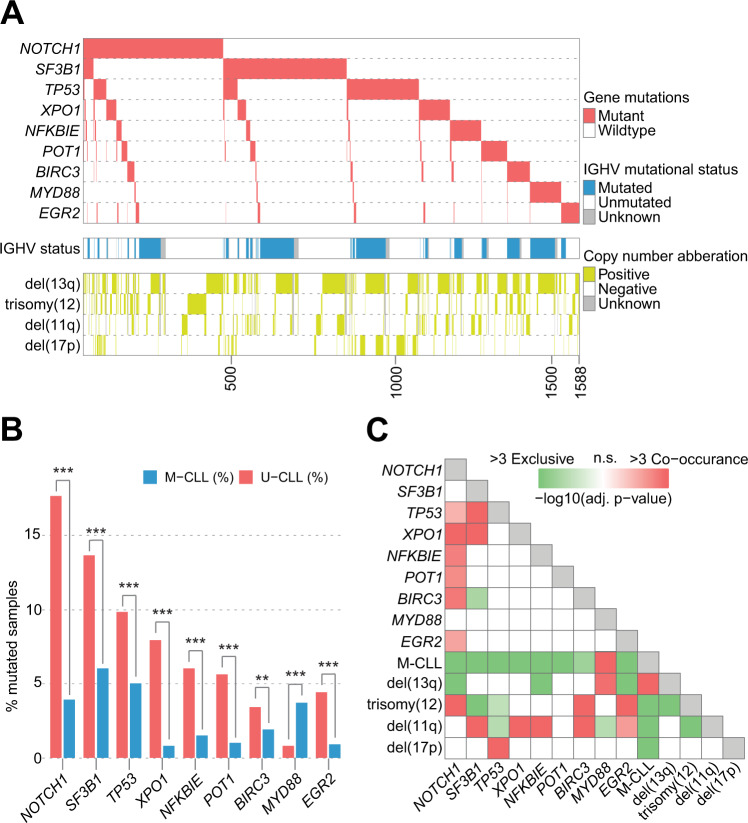
Overview of 1588 CLL cases carrying mutations in recurrently mutated genes. **A** Oncoplot of detected gene mutations (in order of frequency), IGHV gene somatic hypermutation (SHM) status and chromosomal aberrations. **B** Relative distribution of recurrent gene mutations stratified by IGHV gene SHM status, ** denotes a *p* value <0.01, while *** represents a *p* value <0.001. **C** Co-occurence of recurrently mutated genes and chromosomal aberrations.

For the nine genes analyzed, we detected at least one mutation in 1588/4580 (34.7%) patients (Fig. 1A and Supplementary Fig. S1A, B). Of these, mutations were found in a single gene in 1221/1588 (76.9%) patients, while 321 cases carried mutations in 2 genes, and 46 cases had mutations in 3–4 genes. Except for *MYD88*, mutations were significantly more frequent in U-CLL (Fig. 1B), advanced-stage patients (except for *NFKBIE* mutations) and patients requiring treatment (Supplementary Table S2). In contrast, *MYD88* mutations were enriched in M-CLL, while no difference was seen when comparing early-stage versus late-stage patients or patients remaining untreated versus those requiring treatment (Fig. 1A and Supplementary Table S3).

### Clinicobiological profiles associated with recurrent gene mutations

*NOTCH1* mutations were the most common, detected in 448/4580 (9.8%) patients, with the highest frequency among U-CLL (17.6%) of all genes studied (Fig. 1A, B). *NOTCH1*-mutated patients had a relatively high proportion of co-occurring mutations in other genes [181/448 (40.4%) cases], most often in *TP53* (*n* = 45) or *XPO1* (*n* = 41). As most centers performed targeted analysis of exon 34 (86%), the previously reported 2 base-pair frameshift deletion (p.2514fs) [13, 14] was detected in 329/448 (73.4%) mutated samples, while no other hotspot was observed within exon 34 (the locations of all coding mutations identified in all genes are illustrated in Supplementary Fig. S2, while hotspot mutations for all genes are summarized in Supplementary Table S4). As expected, *NOTCH1* mutations were significantly associated with trisomy 12 (Fig. 1C and Supplementary Fig. S3).

*SF3B1* mutations were detected in 428/4580 (9.3%) cases and were the most frequent mutation in M-CLL (148/2454, 6.0%), advanced-stage patients at diagnosis (206/1212, 17.0%) and patients requiring treatment (368/2680, 13.7%). As centers predominantly sequenced exons 14–16 (80%), the majority of mutations (>98%) were localized to these exons, with p.K700E (191/423, 45.2%), p.G742D (54/423, 12.8%) and p.K666E/M/N/Q/R/T (39/423, 9.2%) being the most common hotspot mutations [16, 17, 25, 35–37]. Mutations in other genes were observed in 151/423 (35.7%) *SF3B1*-mutated cases with *TP53* mutations, the most common co-occurring event (50/423, 11.8%). Interestingly, *BIRC3* and *SF3B1* mutations appeared almost mutually exclusive, co-occurring in only 3/423 (0.7%) patients (Fig. 1C). Furthermore, *SF3B1* mutations were negatively associated with trisomy 12, only found in 3.9% of trisomy 12 cases (Fig. 1C and Supplementary Fig. S3 and Supplementary Table S3).

*TP53* mutations were identified in 322/4580 (7.0%) patients with 35/322 (10.9%) cases carrying multiple *TP53* mutations [31/35 (89%) cases carried two mutations, the remaining four cases had three mutations]. Mutations frequently coincided with del(17p) [137/311, 44.1% (no data on genomic aberrations available for 11 cases)] and were the second most frequent mutation in M-CLL [122/2454 (5.0%)] (Fig. 1B, C and Supplementary Fig. S3). *TP53* mutations were detected along the entire coding sequence with p.R248Q/W (*n* = 16) being the most common mutation.

*XPO1* mutations were identified in 176/4580 (3.8%) cases with most mutations detected in U-CLL (88%), patients requiring treatment (90%) and patients carrying mutations in a second gene (97/176, 55.1%). Additional mutations were most commonly found in *NOTCH1* (*n* = 41) or *SF3B1* (*n* = 36) while, on the contrary, only 14 patients carried a second mutation in *TP53*. Patients with *XPO1* mutations often carried del(11q) (20.8% of cases) (Fig. 1A, C and Supplementary Fig. S3). Hotspot mutations at p.E571A/G/K/Q/V were detected in 154/175 (88%, mutation data missing for 1 patient) cases followed by p.D624G detected in 7% of patients.

*NFKBIE* mutations were detected in 150/4580 (3.3%) patients. Among these, 125/150 (83.3%) carried the previously reported frameshift 4-bp deletion (p.Y254fs) [18] while an additional three cases displayed a p.Y254* mutation. Besides *MYD88*, *NFKIBE* was the only other recurrently mutated gene with a similar distribution of mutated cases in early versus late-stage patients, while a significant difference was noted when comparing *NFKBIE* mutations stratified for IGHV gene SHM status or need for treatment (*p* < 0.001 for both comparisons) (Supplementary Table S3).

*POT1* mutations were identified in 142/4580 (3.1%) cases; no mutational hotspot was evidenced with the most common mutations, p.S38C/G/N/R, observed in only 9/142 (6.3%) cases. Most *POT1-*mutated patients showed favorable cytogenetics [isolated del(13q) or no recurrent copy-number aberration (71.9% of all cases); Fig. 1A and Supplementary Fig. S3].

*BIRC3* mutations were observed in 122/4580 (2.7%) cases with 25/122 (20%) carrying the p.Q547fs mutation. The only other mutation found in >5 cases was p.E433fs (*n* = 6). *BIRC3* mutated cases had the highest co-occurrence of del(11q) (35.6% of cases). In fact, a large proportion of patients (70.3%) carrying *BIRC3* mutations harbored unfavorable cytogenetics [trisomy 12, del(11q) or del(17p)] (Fig. 1A and Supplementary Fig. S3).

*MYD88* mutations were detected in 114/4580 (2.5%) patients, with the p.L265P and p.V217F mutations identified in 78/114 (68.4%) and 14/114 (12.3%) cases, respectively. *MYD88* mutations were strongly associated with young age at diagnosis (median 59.9 years, *p* < 0.001) and favorable cytogenetics, with 62.5% of cases carrying only del(13q) (Supplementary Fig. S3). Additionally, *MYD88*-mutated cases rarely [16/114 (14.0%) cases] carried a mutation in a second gene (Fig. 1C).

Finally, *EGR2* mutations were the least common, observed in only 106/4580 (2.3%) cases. Two hotspot mutations were detected; p.H384N found in 56/106 (52.8%) cases, followed by the p.E356K mutation detected in 30/106 (28.3%) cases. *EGR2* mutations were associated with unfavorable cytogenetics (59% of cases) (Fig. 1C and Supplementary Fig. S3 and Supplementary Table S3).

### Recurrent gene mutations and clinical outcome

We assessed the clinical impact of each gene mutation individually using TTFT as the primary endpoint. Patients carrying del(17p) and/or mutations in *TP53* were grouped as *TP53* aberrant (*TP53*ab) as they exhibit a similarly poor outcome [10]: 421 patients were classified as *TP53*ab of whom 44% carried *TP53* mutations, 24% harbored del(17p), while 33% had biallelic *TP53* aberrations.

Except for *MYD88*, all mutated genes were associated with significantly worse outcome (*p* < 0.001); *MYD88*-mutated patients displayed no significant difference in TTFT versus *MYD88* wildtype patients (*p* = 0.092, Supplementary Fig. S4). Multiple *TP53* mutations (excluding del(17p)) had no added effect on TTFT, nor was there a significant difference when comparing patients carrying a *TP53* mutation with or without del(17p). We also investigated if carrying mutations in multiple genes affected clinical outcome. In this analysis, cases harboring *MYD88*-mutations were grouped with cases wildtype for all other genes. Regardless of the gene mutated, patients with ≥2 mutations had a significantly worse TTFT compared to those with a single mutation (*p* < 0.001); while no differences were identified when comparing patients with 2 gene mutations versus those with ≥3 (Supplementary Fig. S5). When stratifying patients based on IGHV gene SHM status, no added effect was found in U-CLL when comparing cases with a single mutation to those with ≥2 mutations. In contrast, multiple mutations were associated with a worse outcome in M-CLL, however, only 65/2454 (2.6%) cases carried mutations in more than 1 gene.

**Fig. 2:**
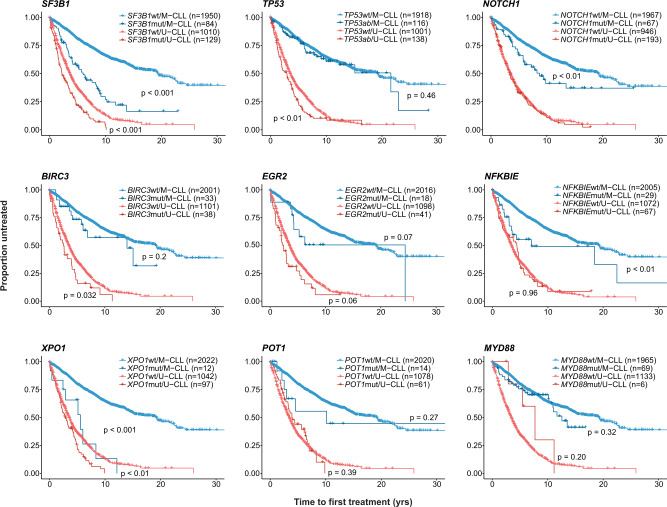
Time-to-first-treatment (TTFT) in Binet A CLL patients carrying recurrent gene mutations stratified based on IGHV gene SHM status. Pairwise comparisons were performed using the Cox–Mantel log-rank test. U-CLL, CLL with unmutated IGHV genes, M-CLL, CLL with mutated IGHV genes.

As advanced-stage disease at diagnosis is an indication for treatment, we performed the analysis in Binet stage A patients and obtained similar results, whereby mutations in all genes, except *MYD88*, resulted in significantly shorter TTFT (*p* < 0.001 for all, Supplementary Fig. S6). We repeated the analysis in the Binet stage A patient cohort, this time stratifying the patients based on IGHV gene SHM status. Notably, only mutations in *SF3B1* and *XPO1* could further discriminate patients with a statistically significant worse outcome in both U-CLL and M-CLL (Fig. 2). *TP53* and *BIRC3* aberrations were significantly correlated with TTFT in U-CLL only, while, *NOTCH1* and *NFKBIE* mutations were significantly associated with shorter TTFT only in M-CLL (Fig. 2). Finally, mutations in *EGR2*, *POT1* and *MYD88* conferred no additional prognostic information to patients stratified by IGHV gene SHM status (Fig. 2).

**Fig. 3:**
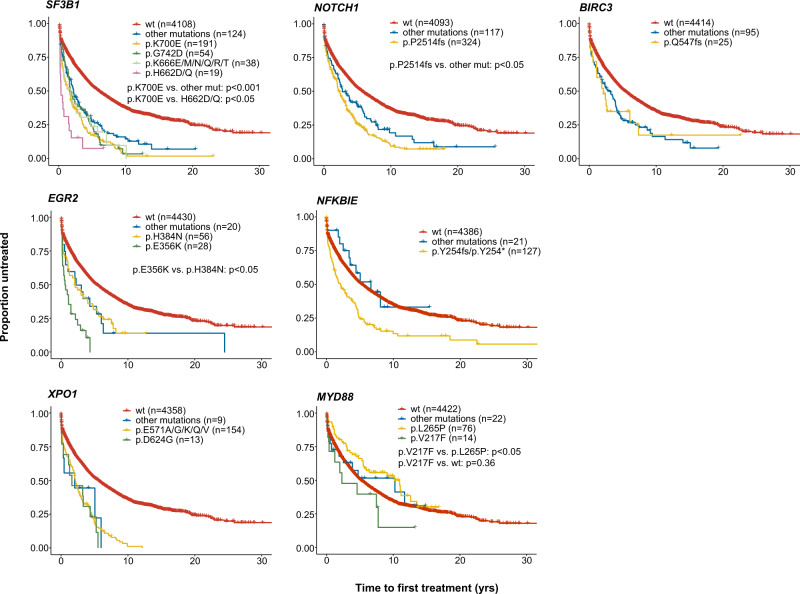
Clinical impact of hotspot versus non-hotspot mutations in recurrently mutated genes assessed using time-to-first-treatment (TTFT) as clinical endpoint. Pairwise comparisons were performed using the Cox–Mantel log-rank test.

We next investigated how hotspot versus non-hotspot mutations within the same gene affected TTFT. As there were no hotspot mutations detected in *TP53* or *POT1*, these genes were omitted from the analysis. Although all non-hotspot *NOTCH1* mutations combined resulted in a worse outcome when compared to *NOTCH1* wildtype patients (*p* < 0.001), cases carrying the hotspot p.2514fs mutation had a particularly poor outcome when compared to non-hotspot mutations (*p* < 0.05, Fig. 3). A similar trend was observed for *SF3B1* mutations whereby patients with hotspot mutations (p.K700E, p.G742D, p.K666E/M/N/Q/R/T, p.H662D/Q and p.I704F/N/S) appeared to have the worst outcome, although the numbers for some hotspot mutations were low (Fig. 3). Among patients with *EGR2* mutations, those carrying the p.E356K mutation showed a tendency toward worse outcome compared to all other *EGR2*-mutated cases, although not statistically significant, while for *NFKBIE*-mutated patients, only the hotspot p.Y254fs mutation significantly affected clinical outcome (Fig. 3). No differences were detected when comparing variants in *XPO1* or *BIRC3*, while for *MYD88* mutated patients, neither hotspot nor non-hotspot mutations differed significantly from wildtype patients (Fig. 3).

### Multivariate analysis reveals different impact of molecular markers in U-CLL and M-CLL

To assess the relationships between the different gene aberrations and their relevance for TTFT in CLL, we first performed a multivariate model focusing on the nine investigated genes, age at diagnosis as well as sex in the entire cohort. As before, *TP53*ab rather than *TP53* mutations were included as a variable and *NFKBIE*-mutated cases were defined as those carrying only the hotspot p.Y254fs/p.Y254* mutation based on the association with poor outcome as described above. All variables other than age at diagnosis and *MYD88* mutations, were found to be significant (Supplementary Table S5). In a second model, which also included IGHV gene SHM status, del(11q), trisomy 12 and Binet stage, *SF3B1*, *TP53*, *EGR2*, *NFKBIE* and *XPO1* mutations remained as significant variables, while *NOTCH1*, *POT1* and *BIRC3* mutations were no longer significant (Supplementary Table S6).

**Fig. 4:**
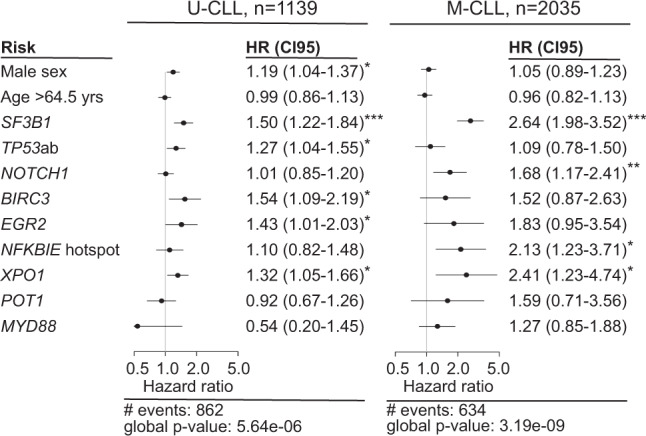
Multivariate analysis in Binet stage A CLL patients with unmutated IGHV genes (U-CLL) and mutated IGHV genes (M-CLL). CI95, 95% confidence interval; * indicates a *p* value <0.05, ** denotes a *p* value <0.01, while *** represents a *p* value <0.001.

Next, we studied Binet A cases only using the same multivariate models and obtained very similar results (Supplementary Tables S5 and S6). We repeated the analysis focusing on the 9 genes (including also age at diagnosis and sex) separately for U-CLL and M-CLL. Notably, *SF3B1* and *XPO1* mutations were the only independent variables in the multivariate analysis of both U-CLL and M-CLL with *SF3B1* showing the highest hazard ratio (Fig. 4). Additionally, male sex, *TP53*, *BIRC3* and *EGR2* aberrations were significant factors for U-CLL, while mutations in *NOTCH1* and *NFKBIE* were significant markers in M-CLL. Using the extended model, including del(11q) and trisomy 12, revealed that *SF3B1* and *XPO1* were again the only independent genes in the multivariate analysis of both U-CLL and M-CLL (Supplementary Table S7).

Finally, by investigating the relative impact of the risk factors associated with TTFT, *SF3B1* mutations was the most significant marker followed by *XPO1* mutations in Binet stage A M-CLL patients, while for Binet stage A U-CLL cases, *SF3B1* mutations was the most significant factor by far (Supplementary Fig. S7).

## Discussion

Over the last 10 years, whole-exome and/or whole-genome sequencing studies have dissected the genomic landscape of CLL, demonstrating that only a few recurrent gene mutations are present in more than 5–10% of cases (*ATM, NOTCH1, SF3B1, TP53*), while the majority are seen in a minor proportion of cases (<1–5%) [20, 21, 32]. To date, recurrent mutations in more than 25 genes have been associated with clinical outcome, most of which affect key signaling pathways and cellular processes [16, 20, 21, 25, 26, 38]. Most of these gene mutations have been linked to a clinically more aggressive disease with shorter TTFT and overall survival. However, while some of these gene mutations have been more comprehensively studied in relatively large cohorts, others are less well explored. Considering the relatively low frequency of mutations in many CLL-related genes, large-scale studies are necessary to offer robust insights into the frequency of these mutations and their impact on prognosis [39].

Another relevant aspect concerns the asymmetric distribution of genomic aberrations between U-CLL and M-CLL, with an enrichment of poor-prognostic alterations in the former [31, 32, 40]. Moreover, the prognostic impact of gene mutations may differ depending on IGHV gene SHM status [29, 31, 32], prompting the suggestion that separate prognostic indices are warranted for U-CLL and M-CLL.

In this study, we analyzed nine CLL-related genes in a cohort of more than 4500 CLL cases to gain comprehensive insight into the impact of different gene mutations on TTFT, particularly in relation to IGHV gene SHM status. The median age at diagnosis was 64.5 years, which is lower than expected and possibly explained by the fact that most contributing institutes are referral centers where patients tend to be younger at admission. As anticipated, a higher prevalence of *NOTCH1*, *SF3B1* and *TP53* aberrations was confirmed, while mutations in the remaining genes were less frequent (between 2–4% of patients). The vast majority of mutated patients carried mutations in a single gene, whereas a minor proportion harbored ≥2 mutations. In line with previous studies, several genes showed prominent mutation hotspots (e.g., *EGR2, MYD88, NFKBIE, BIRC3, NOTCH1, SF3B1, XPO1*), however this was not observed in other genes (e.g., *POT1, TP53*) [18, 19, 41–44]. As previously reported, *NOTCH1* mutations were associated with trisomy 12, *BIRC3* mutations with del(11q) and *MYD88* mutations with sole del(13q) [22, 25, 45, 46]. Notably, certain lesions appeared almost mutually exclusive, such as *BIRC3* and *SF3B1* mutations, and *TP53* aberrations and *XPO1* mutations, implying that acquisition of either of these events is sufficient to develop a more aggressive disease course [47]. For all genes investigated, except for *MYD88*, there was a striking enrichment of mutations in U-CLL compared to M-CLL, particularly for *NOTCH1*, *XPO1, NFKBIE* and *EGR2*. Nevertheless, for all genes investigated, mutations were also detected in a minor proportion of M-CLL patients (1–6% of cases).

In univariate analysis of TTFT, all gene mutations, except for *MYD88*, were associated with a significantly worse outcome, both in the entire study cohort and when restricting the analysis to Binet stage A patients. In multivariate analysis in Binet stage A patients, all recurrently mutated genes, again excepting *MYD88*, were independently associated with shorter TTFT. However, when performing the same analysis in U-CLL and M-CLL separately, only *SF3B1* and *XPO1* mutations retained significance in both subgroups. More specifically, *NOTCH1* and *NFKBIE* mutations were significant only in M-CLL, while *TP53*, *BIRC3* and *EGR2* aberrations were significant only in U-CLL. To evaluate the relative importance of the different gene mutations, we also performed stepwise variable selection in M-CLL and U-CLL separately. This analysis revealed that *SF3B1* and *XPO1* mutations had the highest relative impact on TTFT in M-CLL and *SF3B1* and *TP53* aberrations in U-CLL (Supplementary Fig. 7).

Most previous studies have analyzed the impact of gene mutations in relation to different clinical endpoints (e.g., TTFT, progression-free survival and overall survival) [25, 44, 48]. International prognostic schemes have also been developed to identify patients with high-risk disease (CLL-IPI) or high risk of progression (IPS-E) using similar endpoints [49, 50]. In contrast to these studies, which consider CLL patients independent of IGHV gene SHM status, we recently provided preliminary evidence that different markers were relevant in U-CLL versus M-CLL; i.e., *TP53* abnormalities, del(11q) and/or *SF3B1* mutations in U-CLL and *TP53* abnormalities, trisomy 12 and stereotyped subset #2 membership in M-CLL [31]. In the present study, we confirm and significantly extend these initial findings, further underscoring the varying impact of gene mutations in relation to IGHV gene SHM status. In other words, if the aim is to identify CLL patients with the highest risk of progressive disease and in early need of treatment, different sets of genetic biomarkers should be used in U-CLL and M-CLL. For M-CLL, one should assess *SF3B1, XPO1, NOTCH1* and *NFKBIE*, while *TP53* aberrations do not appear to have any impact on TTFT in this subgroup. Conversely, in U-CLL, *SF3B1, TP53, XPO1*, *BIRC3* and *EGR2* appear to be the most relevant to analyze for identifying high-risk patients.

One of the limitations of our study is the multicenter data collection, where different sequencing techniques/targeted approaches have been applied with varying sensitivity. That said, in a recent multicenter study performed by ERIC, high concordance was observed for NGS-based gene panels above a VAF of 5% or more, a cutoff which we also applied in the present study [51]. For a minor proportion of cases, approaches that only targeted hotspot mutations [e.g., *NFKBIE* (8% of cases) and *MYD88* (4% of cases)], or hotspot exons (e.g., *SF3B1* and *NOTCH1*) could potentially underestimate the true frequency of mutations. Here, we found no significant differences in mutation rates when comparing NGS and Sanger sequencing for either hotspot or non-hotspot genes. Additionally, in the case of *NFKBIE*, only the hotspot mutations were clinically relevant, whereas for the remaining genes the significance, if any, of non-hotspot mutations remains unknown. However, we cannot fully exclude that mutations with low frequencies (VAF 5–15%) were missed using Sanger sequencing. In addition, the majority of samples (>80%) derived from non-purified PBMC samples (<20% of samples were purified), which could potentially have affected the ability to detect minor subclones, although the vast majority of unpurified CLL samples have a high tumor percentage (>80–90%). Another caveat concerns the differences in treatment given at the different centers, precluding a meaningful analysis of the impact of gene mutations on overall survival. Moreover, most patients have received chemo(immuno)therapy, hence, in the coming years it will be important to conduct similar large-scale, real-world analysis of the impact of treatment in patients using targeted treatments.

In conclusion, we reinforce *SF3B1* mutations as a key biomarker with a very strong negative impact on TTFT in both M-CLL and U-CLL and also highlight *XPO1* as an additional highly relevant gene in both subgroups. We also demonstrate that *TP53* aberrations are clinically relevant in U-CLL, yet they appear to have no or a limited effect on TTFT in M-CLL. From a clinical perspective, our results may assist in identifying high-risk patients within the heterogeneous M-CLL subgroup with potential implications for stratified management and treatment decisions, also in prospective clinical trials. Since most clinical laboratories are currently applying NGS-based gene panel sequencing to detect *TP53* mutations, often including other CLL-related genes (that are usually not reported), it would be informative to extend the analysis to other genes to identify high-risk patients in routine diagnostics. Finally, future efforts to develop prognostic schemes including gene mutations and other established prognostic factors should apply a more compartmentalized approach, hopefully paving the way for personalized medicine approaches for patients belonging to the M-CLL and U-CLL subgroups.

## Supplementary information

Supplemental information

Supplemental Tables

## Data Availability

The data that support the findings of this study are available from the corresponding author upon reasonable request.
