# Correction: Different prognostic impact of recurrent gene mutations in chronic lymphocytic leukemia depending on IGHV gene somatic hypermutation status: a study by ERIC in HARMONY

**DOI:** 10.1038/s41375-023-01813-3

**Published:** 2023-01-12

**Authors:** Larry Mansouri, Birna Thorvaldsdottir, Lesley-Ann Sutton, Georgios Karakatsoulis, Manja Meggendorfer, Helen Parker, Ferran Nadeu, Christian Brieghel, Stamatia Laidou, Riccardo Moia, Davide Rossi, Mark Catherwood, Jana Kotaskova, Julio Delgado, Ana E. Rodríguez-Vicente, Rocío Benito, Gian Matteo Rigolin, Silvia Bonfiglio, Lydia Scarfo, Mattias Mattsson, Zadie Davis, Ajay Gogia, Lata Rani, Panagiotis Baliakas, Hassan Foroughi-Asl, Cecilia Jylhä, Aron Skaftason, Inmaculada Rapado, Fatima Miras, Joaquín Martinez-Lopez, Javier de la Serna, Jesús María Hernández Rivas, Patrick Thornton, María José Larráyoz, María José Calasanz, Viktória Fésüs, Zoltán Mátrai, Csaba Bödör, Karin E. Smedby, Blanca Espinet, Anna Puiggros, Ritu Gupta, Lars Bullinger, Francesc Bosch, Bárbara Tazón-Vega, Fanny Baran-Marszak, David Oscier, Florence Nguyen-Khac, Thorsten Zenz, Maria Jose Terol, Antonio Cuneo, María Hernández-Sánchez, Sarka Pospisilova, Ken Mills, Gianluca Gaidano, Carsten U. Niemann, Elias Campo, Jonathan C. Strefford, Paolo Ghia, Kostas Stamatopoulos, Richard Rosenquist

**Affiliations:** 1 Department of Molecular Medicine and Surgery, Karolinska Institutet, Stockholm, Sweden; 2 Centre for Research and Technology Hellas, Institute of Applied Biosciences, Thessaloniki, Greece; 3 Department of Mathematics, University of Ioannina, Ioannina, Greece; 4 MLL Munich Leukemia Laboratory, Munich, Germany; 5 Cancer Genomics, School for Cancer Sciences, Faculty of Medicine, University of Southampton, Southampton, UK; 6 Institut d’Investigacions Biomèdiques August Pi i Sunyer (IDIBAPS), Barcelona, Spain; 7 Centro de Investigación Biomédica en Red de Cáncer (CIBERONC), Madrid, Spain; 8 Department of Hematology, Rigshospitalet, Copenhagen University Hospital, Copenhagen, Denmark; 9 Division of Hematology, Department of Translational Medicine, Università del Piemonte Orientale, Novara, Italy; 10 Division of Hematology, Oncology Institute of Southern Switzerland, Bellinzona, Switzerland; 11 Laboratory of Experimental Hematology, Institute of Oncology Research, Bellinzona, Switzerland; 12 Patrick G Johnston Centre for Cancer Research, Queen’s University Belfast, Belfast, UK; 13 Department of Internal Medicine—Hematology and Oncology, University Hospital Brno, Brno, Czech Republic; 14 Faculty of Medicine, Masaryk University, Brno, Czech Republic; 15 Central European Institute of Technology, Masaryk University, Brno, Czech Republic; 16 Hospital Clínic of Barcelona, Barcelona, Spain; 17 Universitat de Barcelona, Barcelona, Spain; 18 Cancer Research Center (IBMCC) CSIC—University of Salamanca, Salamanca, Spain; 19 Instituto de Investigación Biomédica (IBSAL), Salamanca, Spain; 20 Department of Hematology, University Hospital of Salamanca, Salamanca, Spain; 21 Hematology—Department of Medical Sciences, University of Ferrara, Ferrara, Italy; 22 Università Vita Salute San Raffaele and IRCCS Ospedale San Raffaele, Milano, Italy; 23 Department of Immunology, Genetics and Pathology, Uppsala University, Uppsala, Sweden; 24 Molecular Pathology Department, University Hospitals Dorset, Bournemouth, UK; 25 All India Institute of Medical Sciences (AIIMS), New Delhi, India; 26 Hospital Universitario 12 Octubre, Madrid, Spain; 27 Spanish National Cancer Research (CNIO), Madrid, Spain; 28 Haematology Department, Beaumont Hospital, Dublin, Ireland; 29 Hematological Diseases Laboratory, CIMA LAB Diagnostics, University of Navarra, Pamplona, Spain; 30 IdiSNA, Navarra Institute for Health Research, Pamplona, Spain; 31 HCEMM-SE Molecular Oncohematology Research Group, Department of Pathology and Experimental Cancer Research, Semmelweis University, Budapest, Hungary; 32 Central Hospital of Southern Pest—National Institute of Hematology and Infectious Diseases, Budapest, Hungary; 33 Clinical Epidemiology Division, Department of Medicine Solna, Karolinska Institutet, Stockholm, Sweden; 34 Molecular Cytogenetics Laboratory, Pathology Department, Hospital del Mar and Translational Research on Hematological Neoplasms Group, Hospital del Mar Research Institute (IMIM), Barcelona, Spain; 35 Department of Hematology, Oncology and Cancer Immunology, Charité-Universitätsmedizin Berlin, Corporate Member of Freie Universität Berlin, Humboldt-Universität zu Berlin, Berlin, Germany; 36 Department of Hematology, Hospital Universitari Vall d’Hebron (HUVH), Experimental Hematology, Vall d’Hebron Institute of Oncology (VHIO), Department of Medicine, Universitat Autònoma de Barcelona, Barcelona, Spain; 37 Service d’hématologie Biologique Hôpital Avicenne Assistance Publique des Hôpitaux de Paris, Bobigny, France; 38 Sorbonne Université, Service d’Hématologie Clinique, Hôpital Pitié-Salpêtrière, APHP, Paris, France; 39 Department of Oncology and Haematology, University Hospital and University of Zurich, Zurich, Switzerland; 40 Department of Hematology, INCLIVA Research Insitute, University of Valencia, Valencia, Spain; 41 Clinical Genetics, Karolinska University Hospital, Solna, Sweden

**Keywords:** Cancer genetics, Genetics research

Correction to: *Leukemia* 10.1038/s41375-022-01802-y, published online 24 December 2022

In this article the author name Florence Nguyen-Khac was incorrectly written as Florence N’Guyen-Khac.

The original article has been corrected.

