# Supplementary material for: Different prognostic impact of recurrent gene mutations in chronic lymphocytic leukemia depending on IGHV gene somatic hypermutation status: a study by ERIC in HARMONY
Source: Leukemia. 2022 Dec 24;37(2):339–47. doi: 10.1038/s41375-022-01802-y (PMC9898037; doi:10.1038/s41375-022-01802-y)
Supplement: Supplementary file 1 — Supplemental information [file 41375_2022_1802_MOESM1_ESM.docx]

**Supplemental data**

**Different** **prognostic impact of gene mutations in CLL with mutated or unmutated immunoglobulin genes: A study by ERIC in HARMONY**

Mansouri *et. al.*

**Supplemental Figures:**


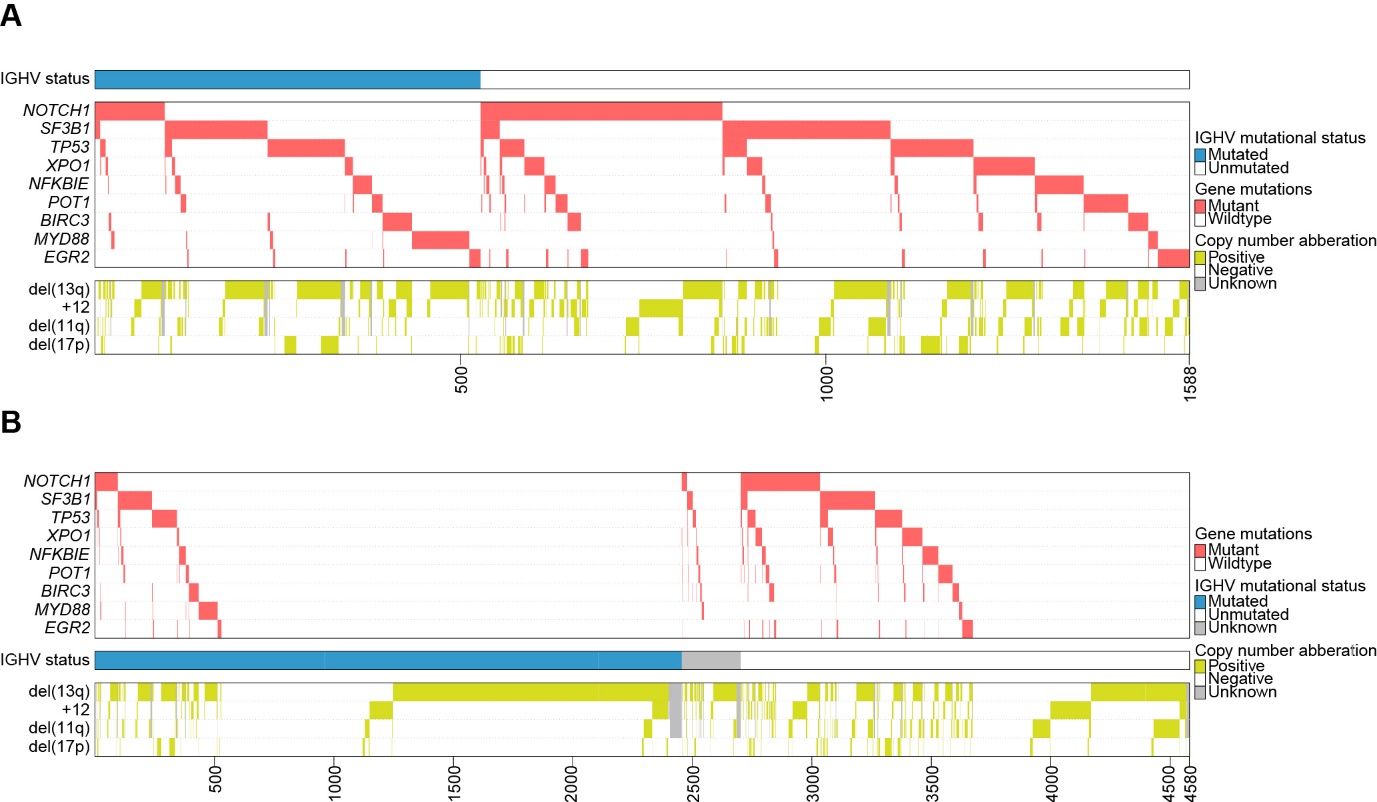


**Supplemental Figure S1.** (A) Overview of 1588 CLL cases carrying mutations in recurrently mutated genes sorted by IGHV gene somatic hypermutation status and (B) in all 4,580 CLL cases sorted by IGHV gene somatic hypermutation status.


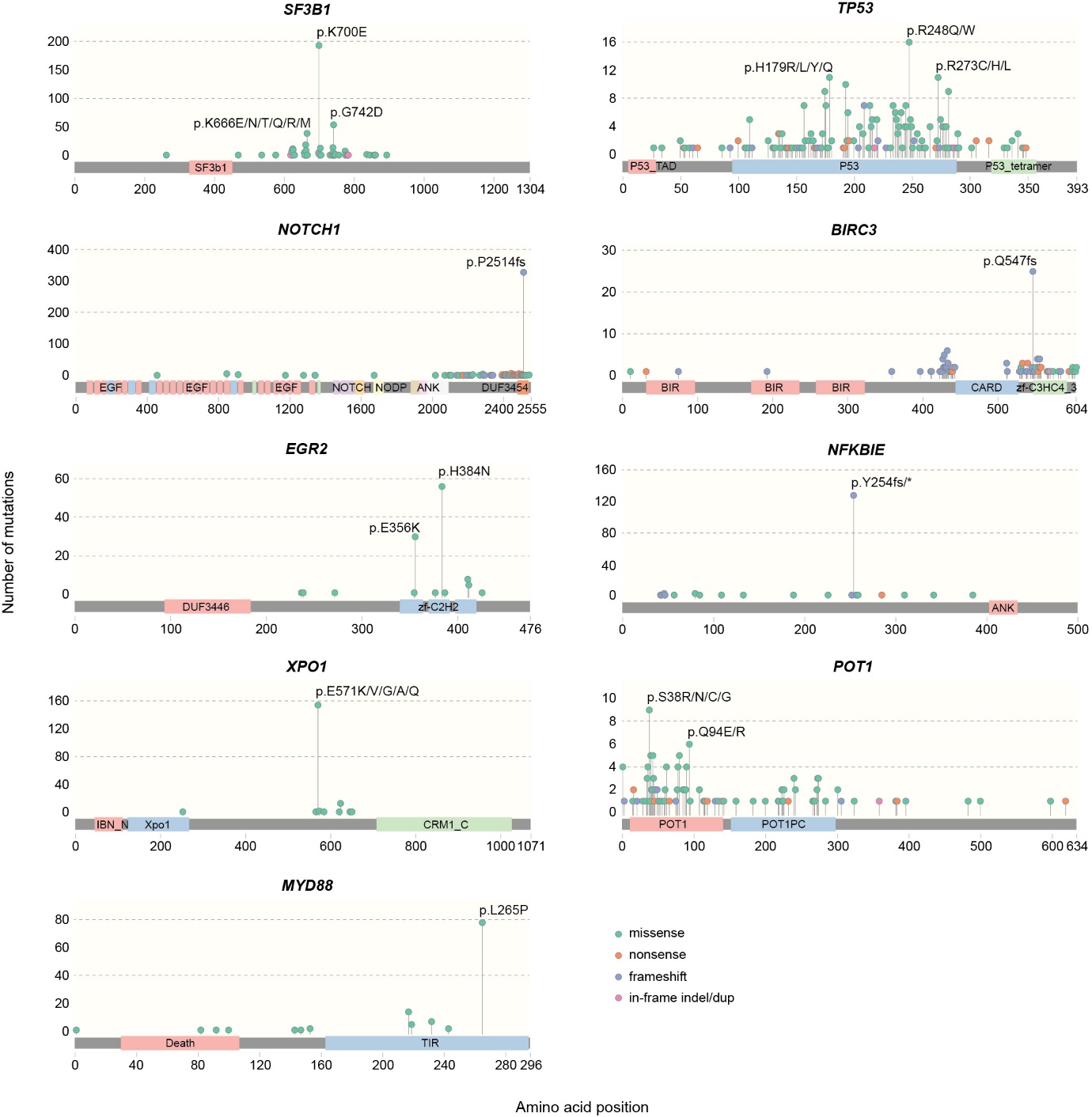


**Supplemental Figure S2.** Graphical representation of gene mutations and the resulting amino acid change for all CLL genes studied.


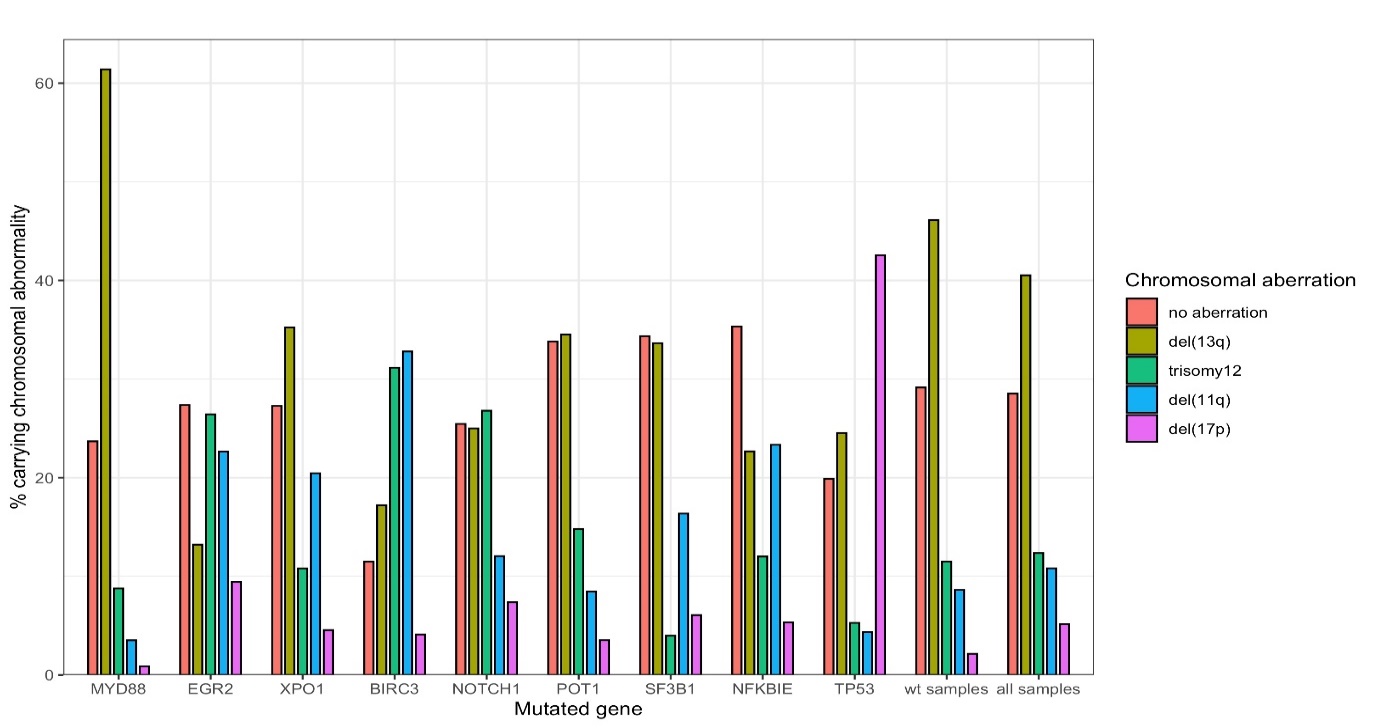


**Supplemental Figure S3.** Distribution of chromosomal aberrations in patient carrying mutations in recurrently mutated genes.


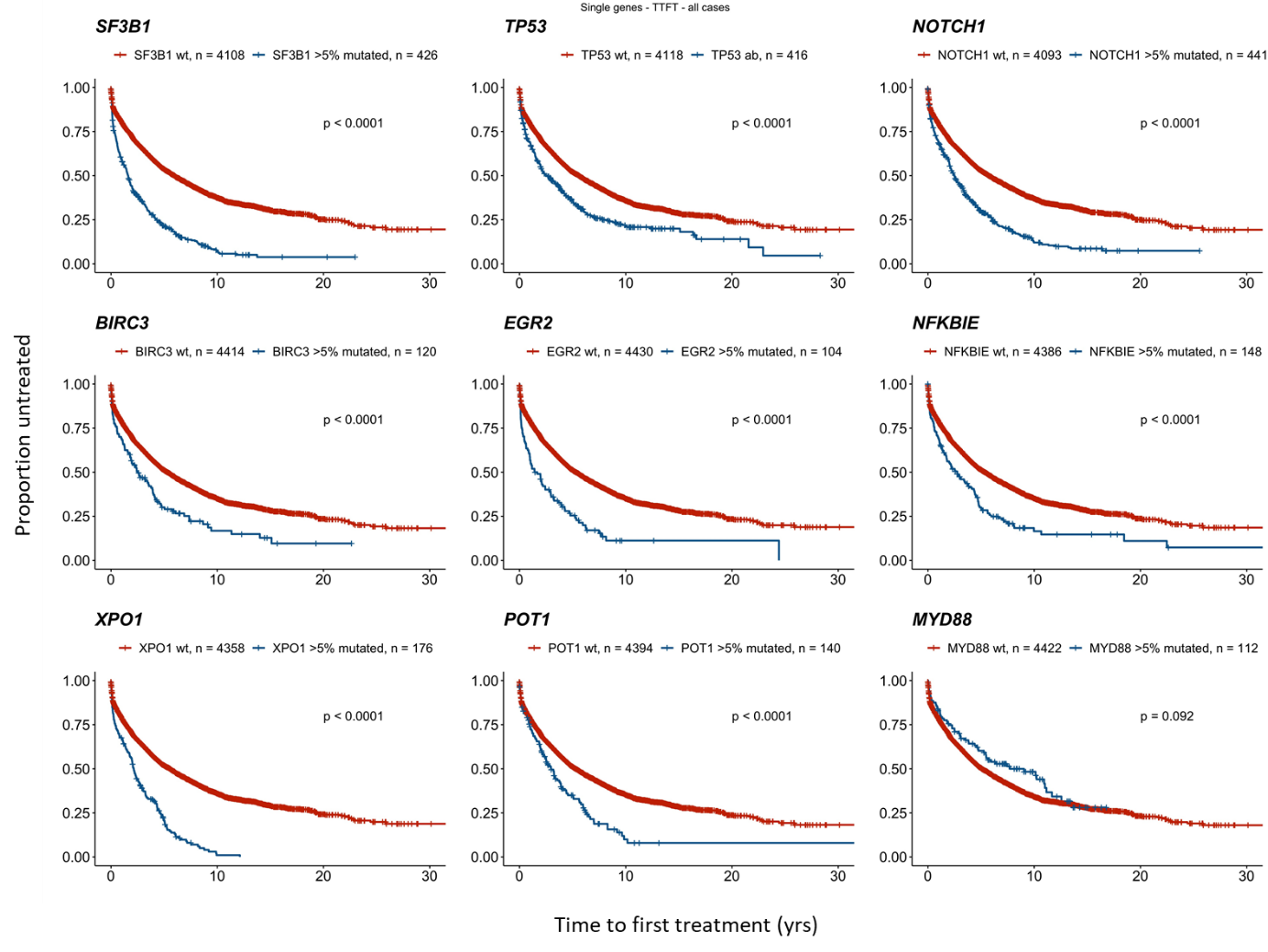


**Supplemental Figure S4.** Time-to-first-treatment in all CLL patients carrying recurrent gene mutations.


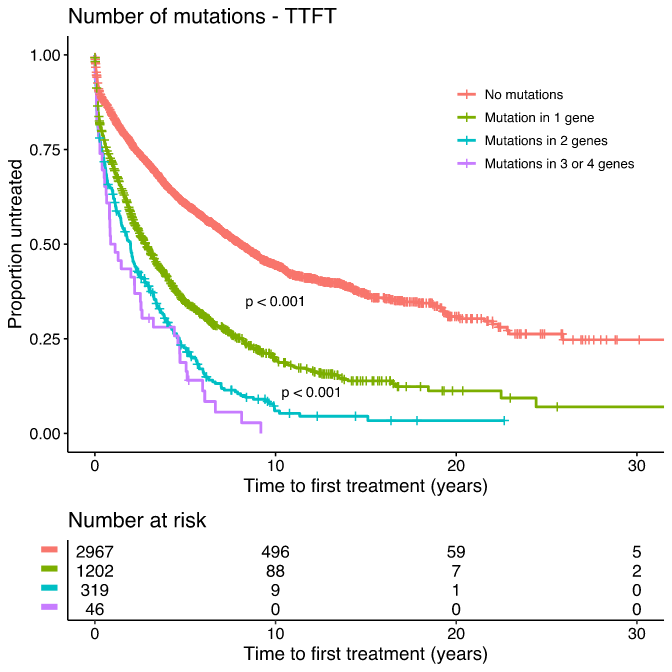


**Supplemental Figure S5.** Multiple mutations and clinical outcome.


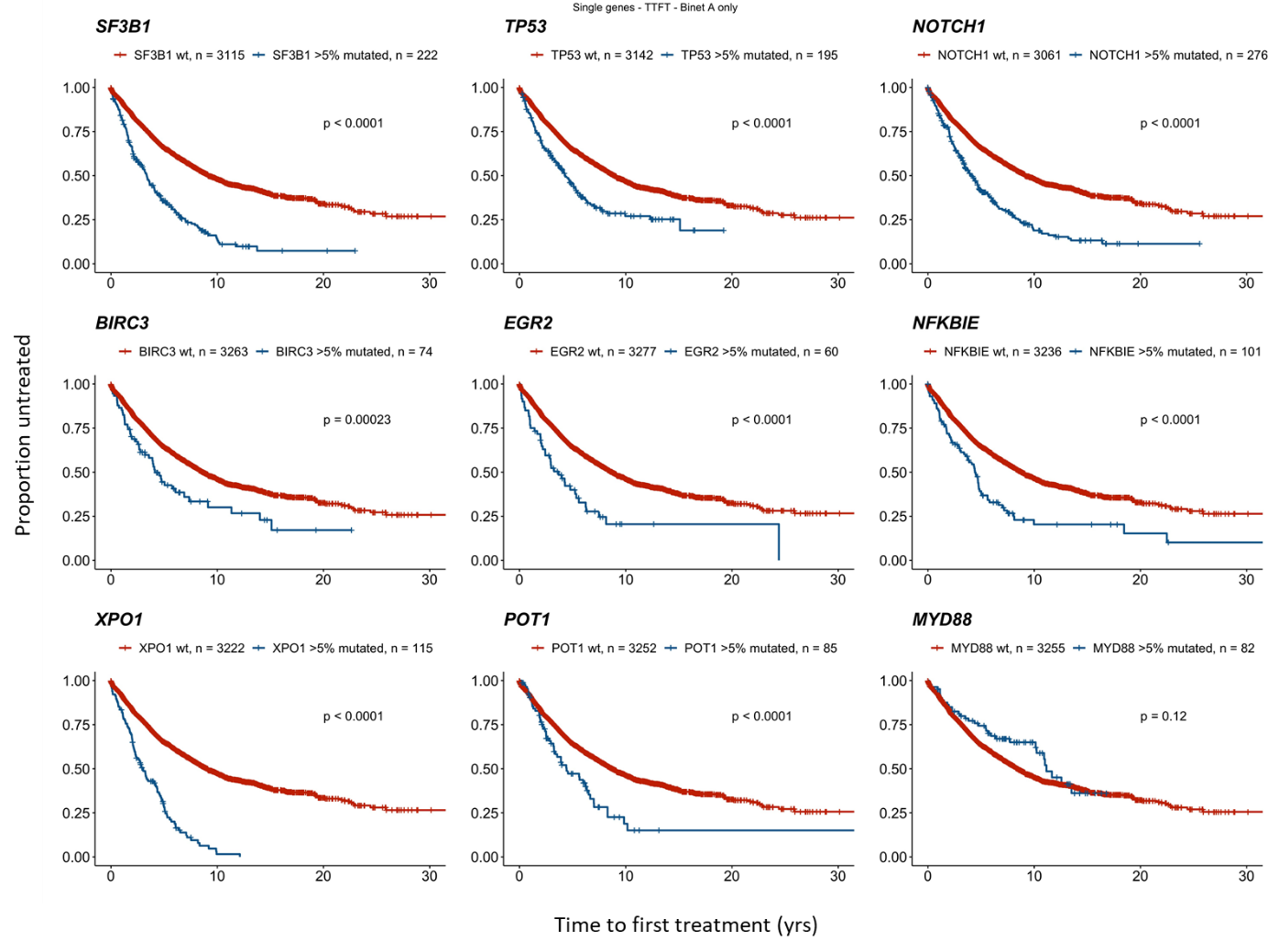


**Supplemental Figure S6.** Time-to-first-treatment in Binet A CLL patients carrying recurrent gene mutations.


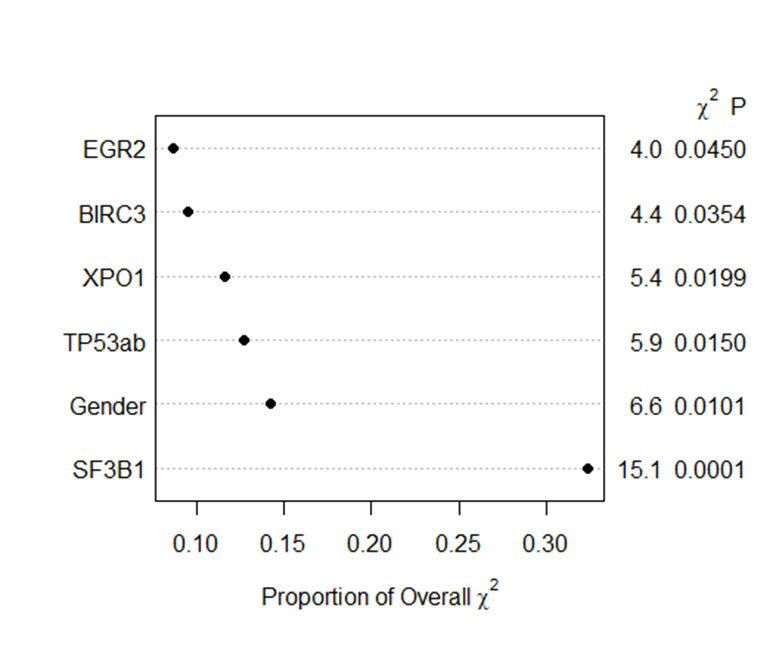

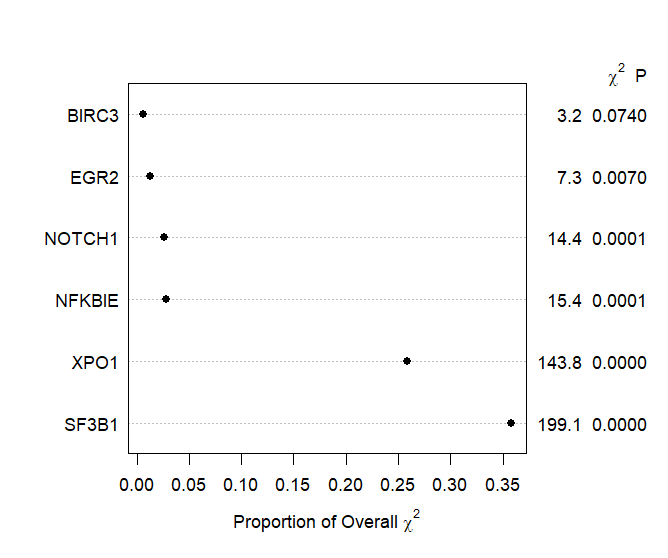


A

B

C

D

**Supplemental Figure S7.** Reduced Cox regression model and relative importance of the risk factors for Binet A CLL in (A-B) IGHV gene mutated and (C-D) IGHV gene unmutated patients using stepwise variable selection.
